# Potential of Natural Phenolic Compounds against Doxorubicin-Induced Chemobrain: Biological and Molecular Mechanisms Involved

**DOI:** 10.3390/antiox13040486

**Published:** 2024-04-18

**Authors:** Simona Serini, Gabriella Calviello

**Affiliations:** 1 Department of Translational Medicine and Surgery, Section of General Pathology, School of Medicine and Surgery, Università Cattolica del Sacro Cuore, Largo F. Vito, 00168 Rome, Italy; 2 Fondazione Policlinico Universitario A. Gemelli IRCCS, Largo F. Vito, 00168 Rome, Italy

**Keywords:** phenolic compounds, antioxidant, cognitive impairment, neurotoxicity, oxidative stress

## Abstract

Chemotherapy-induced cognitive impairment or “chemobrain” is a prevalent long-term complication of chemotherapy and one of the more devastating. Most of the studies performed so far to identify the cognitive dysfunctions induced by antineoplastic chemotherapies have been focused on treatment with anthracyclines, frequently administered to breast cancer patients, a population that, after treatment, shows a high possibility of long survival and, consequently, of chemobrain development. In the last few years, different possible strategies have been explored to prevent or reduce chemobrain induced by the anthracycline doxorubicin (DOX), known to promote oxidative stress and inflammation, which have been strongly implicated in the development of this brain dysfunction. Here, we have critically analyzed the results of the preclinical studies from the last few years that have evaluated the potential of phenolic compounds (PheCs), a large class of natural products able to exert powerful antioxidant and anti-inflammatory activities, in inhibiting DOX-induced chemobrain. Several PheCs belonging to different classes have been shown to be able to revert DOX-induced brain morphological damages and deficits associated with learning, memory, and exploratory behavior. We have analyzed the biological and molecular mechanisms implicated and suggested possible future perspectives in this research area.

## 1. Introduction

Chemotherapy often induces considerable short-term alterations in the functions of multiple organ systems (such as bone marrow, mouth, digestive tract, etc.), which sometimes represent major obstacles to continuing anticancer treatments [1]. Besides these early adverse effects, chemotherapeutic treatments may also induce additional detrimental consequences for health in the long term, which may result in sustained extensive changes in the quality of life. Particularly, among chemotherapy-induced long-term adverse effects, chemotherapy-induced cognitive impairment is considered one highly prevalent complication of chemotherapy and one of the most devastating for its symptomatology, since it may last throughout the lifetime in a considerable portion of survivors [2,3]. This peculiar form of cognitive impairment is also referred to with more colloquial terms such as “chemobrain” or “chemofog” and includes memory loss and difficulty in performing several tasks, such as those related to speech, learning, concentration, and other psychomotor functioning [4,5,6,7,8,9]. Even though the affected patients have always reported their chemobrain-related symptomatology to health professionals, it is quite surprising that it has had scarce relevance for decades, probably since it has been generally thought to be an obvious consequence of the stress related to cancer diagnosis and treatments [10]. This interpretation was probably related to the misconception that chemotherapeutic agents are usually not able to cross the blood–brain barrier (BBB) [10,11]. It is only in the past two decades that this peculiar form of cognitive impairment has started to be given due attention by researchers, with the aims of better defining it, as well as finding possible strategies for its prevention [10,12]. Therefore, it has been recognized that a large portion of cancer patients develop this form of cognitive impairment after chemotherapy, even though the rates reported in a variety of studies and meta-analyses differ markedly in relation to the age and type of cancer or therapy [13]. Most of these studies have been performed on breast cancer (BCa) patients, especially old patients, as the possibility of survival for many years is very high in these patients due to the advances in BCa treatments [9]. Thus, in a very recent systematic review evaluating the prevalence of this cognitive dysfunction in old BCa patients, it was observed that the estimated prevalence ranged from 18.6% to 27% and from 7.6 to 49% depending on the method of assessment used (objective neuropsychological tests or subjective cognitive assessments) [9]. Other papers, however, reported higher levels of prevalence, reaching maximum values of 75–78% [3,14,15].

In recent years, a large body of literature has been dedicated to identifying new ways to prevent the toxic effects of chemotherapeutics on the brain and the consequent development of chemobrain, with the final aim of substantially improving the quality of life of patients [16,17,18]. However, it should be considered that, among the chemotherapeutic agents commonly used for treating a variety of cancers, many are able to induce chemobrain, such as cyclophosphamide (CP) [18], mitoxantrone [19], 5-fluorouracil [20], cisplatin [21], paclitaxel [22], and the anthracyclines [23]. However, the mechanisms thought to underline their neurotoxic effects rarely overlap, being often different and specific for each single drug [7]. Most of the studies performed so far on the cognitive dysfunction related to antineoplastic chemotherapy were focused on treatments with anthracyclines and, in particular, doxorubicin (DOX), which is one of the drugs most frequently administered to BCa patients, a population particularly studied for the neurotoxic long-term effect of chemotherapy [6,9]. On this basis, we decided to critically analyze the results of papers published in the last eight years (2016–2024) and focused on identifying possible strategies against DOX-induced chemobrain. This drug exerts prevalent pro-oxidant activity [24] and, through the oxidative stress induced in tissues, is also able to exert pro-inflammatory activity. On this basis, in search of a safe strategy for protecting neoplastic patients from DOX-induced neurotoxicity, a series of preclinical studies have been performed to test the potential of phenolic compounds (PheCs), a large class of natural dietary products showing the main common characteristics (besides the affinity in the chemical structure) of exerting powerful antioxidant and anti-inflammatory activities. Thus, we thought to restrict our analysis to the results obtained so far on the possible protective activity of these compounds against DOX-induced chemobrain. It is interesting to underline that PheCs themselves have been also shown to possess antineoplastic properties, and, for this reason, their constant intake is considered an important strategy for the prevention of several kinds of cancer, including BCa [25,26]. Furthermore, we know that combinatory strategies with multiple antineoplastic chemotherapeutics are largely and more successfully used nowadays against many kinds of cancer as compared to single-agent therapies [27]. In this regard, it is interesting to consider that there are also plenty of results demonstrating that combining anticancer therapies with natural products that have antineoplastic activities and, in particular, anthracyclines with PheC treatments may allow a reduction in the doses of the drugs used and their toxicity, while obtaining improved anticancer effects [28,29,30].

Finally, however, it seems necessary to clarify that focusing on a particular type of chemotherapy and its neurotoxic effects, as well as on the possible strategies to avoid them, should not be considered outdated. In fact, nowadays, some may think that the use of chemotherapy is destined to disappear and thus, any effort expended in studying the way to improve its outcomes could appear quite inappropriate, given that more advanced forms of antineoplastic therapies are now available, such as targeted therapy or immunotherapy. However, this is not completely true, since chemotherapy in general, and particularly that carried out with anthracyclines, still represents the best option for a large number of patients in many cases. As a matter of fact, these drugs are still among the most frequently administered ones when we consider therapy for hematologic tumors, as well as for a large number of solid tumors including lung, breast, ovarian, bladder, thyroid, gastric cancer and neuroblastoma [31]. Moreover, recent observations have shown that chemotherapeutics act not only by exerting direct cytotoxic activity on highly proliferating cancer cells but also by inducing immunogenic cell death [32,33]. This means that these drugs have the capacity to strengthen the immune response that is naturally, but ineffectively, carried out against tumors. It has also been seen that, due to this capacity, chemotherapy is able to prolong the beneficial effects of immunotherapy when combined with it [34,35]. These recent advancements in knowledge underline that innovating chemotherapy to make it more effective and safer for patients still represents an important objective to be pursued.

## 2. DOX: Chemotherapeutic Activity and Mechanisms Underlying Its Toxic Effect at Brain Level

**Figure 1:**
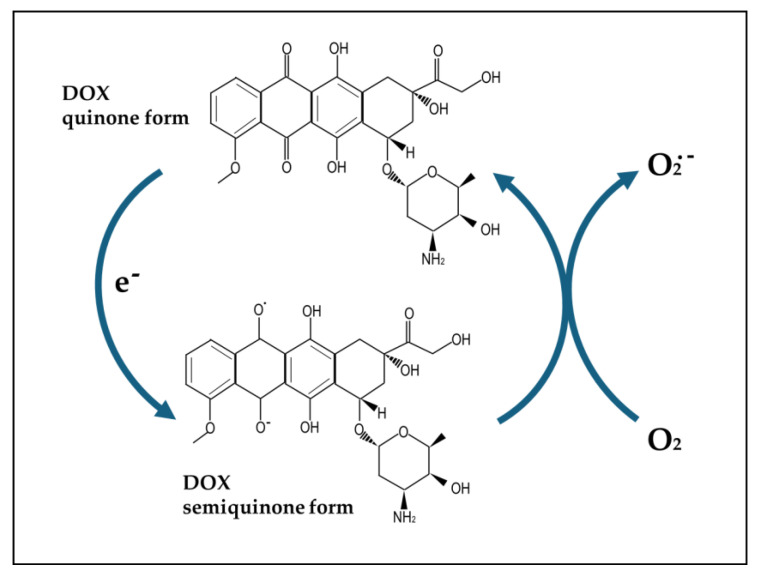
Doxorubicin (DOX) quinone and semiquinone moieties.

DOX is among the most-studied drugs in papers focusing on chemobrain [36]. It is a powerful and widely used drug belonging to the class of anthracyclines, and it was first isolated from the bacteria *Streptomyces* [37]. It is used as a chemotherapeutic agent in a variety of tumors [38], being able to exert strong cytotoxic effects, especially in highly proliferating cells. The main mechanisms underlying its anticancer and cytotoxic action are related to its ability to intercalate between base pairs and, thus, alter DNA metabolism and RNA synthesis, as well as block DNA replication and transcription and induce strand breaks by inhibiting the activity of the enzyme topoisomerase II [39,40]. Moreover, due to the presence of a quinone in its moiety, DOX undergoes redox cycling, which leads to the generation of large amounts of ROS able to induce cell damage and death (Figure 1). Furthermore, additional mechanisms underlying the anticancer activity of this drug have recently been identified, such as the induction of senescence, autophagy, pyroptosis, and ferroptosis, as well as its capacity to modulate the anti-tumor immune response [24,41,42,43].

We know that different chemotherapeutics induce different side effects through diverse mechanisms [6,8]. In particular, DOX has been extensively studied for many adverse effects, above all cardiotoxicity [44], but also damage to many other organs including the liver, kidney, and brain [45,46,47]. We will not discuss the specific mechanisms through which DOX induces neurotoxicity and chemobrain here in detail, since several recent reviews of the literature have exhaustively analyzed and discussed this subject [5,6,7,8].

**Figure 2:**
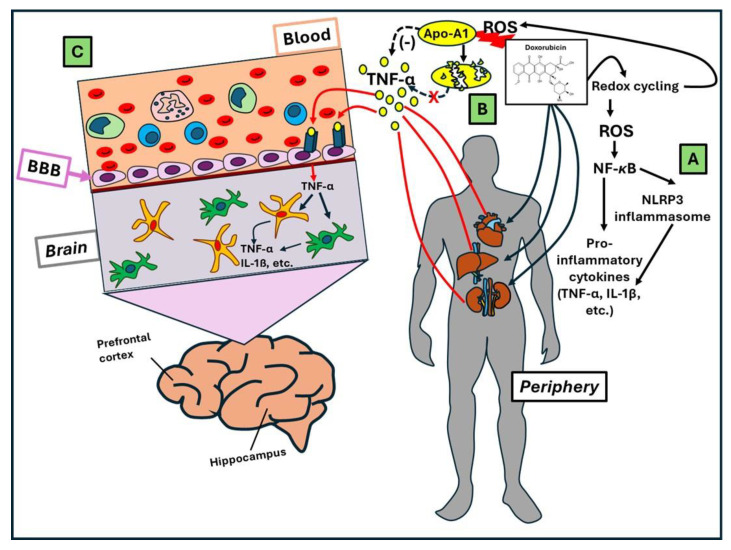
**Indirect mechanisms of DOX-induced chemobrain.** The most-recognized indirect mechanisms underlying DOX-induced neurotoxicity and chemobrain are illustrated. (A) Through redox cycling, DOX generates an excessive amount of ROS, which induce oxidative stress in peripheral tissues. As a consequence, ROS-induced activation of the transcription factor NF-κB and an increase in the NF-κB-dependent transcription of pro-inflammatory cytokines (such as TNF-α or pre-IL-1β) are observed [59]. NF-κB has also been reported to induce the activation of the NLRP3 inflammasome in peripheral cells after treatment with DOX [65]. (B) ROS also produce oxidation and destruction of apolipoprotein A1 (Apo-A1), a negative regulator of TNF-α expression, thus further increasing TNF-α levels in the circulation [64]. (C) It was suggested that TNF-α may enter the brain through its binding to specific receptors located in the BBB [62,63]. Once inside the brain, it may induce innate and adaptative immune cells, as well as brain cells, to further produce pro-inflammatory cytokines, including TNF-α.

Nevertheless, we briefly consider some aspects of DOX-induced neurotoxicity that cannot be ignored to better comprehend the healthy effect that treatments with PheCs can exert on the DOX-induced chemobrain. First, it is important to mention here that the areas that have been reported to be mainly affected by DOX in the brain are the prefrontal cortex (PFC) and the hippocampus (HIP). Their damage is directly connected to the development of deficits associated with learning and memory, as well as exploratory behavior [48]. Moreover, it is worth underlining that the pathogenesis of DOX-induced neurotoxicity has been related to either direct effects of very small amounts of this drug that manage to cross the BBB or indirect mechanisms. Actually, DOX shows a very limited capacity to directly reach the brain and, for this reason, different strategies have been explored to make the delivery of adequate amounts of this drug to intracerebral tumors possible, for instance, by enclosing it in nanoparticles able to cross the BBB and specifically reach cancer cells located inside the brain [49,50]. This means that most of the neurotoxic activity of this drug is thought to be exerted through indirect mechanisms. In any case, according to what has been obtained in plenty of in vitro studies, the small amount of DOX that succeeds in crossing the BBB, once inside the brain, has the potential to induce many alterations in neurons, such as crosslinking of DNA and the following production of DNA double-strand breaks, as well as alterations in DNA repair mechanisms and the induction of apoptosis [47,51]. The DOX-induced ROS production at the brain level has also been suggested as a major mechanism of cytotoxicity and is strictly related to the induction of apoptosis [52]. DOX-induced morphological alterations in neuronal cells have also been reported by in vitro studies, such as DOX-induced neuronal cell damage and death with prevailing chromatin condensation and cell membrane alterations, as well as decreased neurite numbers and reduced differentiation [53]. However, as considered above, the indirect mechanisms of central neurotoxicity, such as inflammation and oxidative stress, seem to be prevalent and are widely supported by plenty of in vivo studies [54,55,56]. It is quite intuitive that, due to the above-mentioned ability of DOX to generate an excessive amount of ROS, this may also increase oxidative stress in peripheral tissues, which, differently from the brain, are easily reached and directly affected by DOX. In turn, ROS may modify the levels and expression of antioxidants and cause oxidation and damage to crucial cellular macromolecules such as lipids, proteins, and nucleic acids [57,58]. Moreover, increased levels of ROS may induce inflammation through the activation of the nuclear transcription factor NF-κB, which is known to activate the transcription of pro-inflammatory cytokines [59] (Figure 2). Accordingly, the level of the pro-inflammatory cytokine TNF-α was found to be increased in the circulation of patients following the i.p. administration of DOX [60]. Moreover, when the same treatment was performed in animals, it was possible to observe that this cytokine increased not only in the circulation but also at the brain level [53,61]. It was suggested that a route for circulatory TNF-α to enter the brain is its binding to specific receptors located in the BBB [62,63]. Then, once inside the brain, TNF-α may induce innate and adaptative immune cells, as well as brain cells themselves, to further produce pro-inflammatory cytokines, including TNF-α. Interestingly, another possibility suggested for explaining the DOX-induced increase in the levels of TNF-α was related to the discovery that one specific target of DOX-induced oxidation and destruction is apolipoprotein A1 (Apo-A1), which was found to act as a negative regulator of TNF-α expression [64]. Moreover, it has also been recently observed that DOX is able to induce the expression of the NLRP3 inflammasome, IL-1, and IL-18 in cardiac cells in vitro [65] and increase the levels of IL-1 and IL-18 in the serum of animals treated with DOX and showing cardiotoxicity [66]. Interestingly, it was recently found that, in the prefrontal cortical tissue of DOX-treated rats, the protein expression of NLRP3, caspase-1, and IL-1β was increased [67]. A similar increase in the protein components of the NLRP3 system was also recently confirmed in the HIP of rats treated with a combination of DOX and CP [68]. Altogether, these results suggest that the activation of the NLRP3 inflammasome may represent a mechanism of DOX-induced chemobrain [69].

## 3. PheCs: Natural Products with Antioxidant, Anti-Inflammatory and Antineoplastic Activities

PheCs are a class of compounds widely distributed in plants and characterized by the presence of one or more phenol rings in their molecular structure. They represent important components of food, having relevant nutritional properties and contributing to the organoleptic characteristics of foods, such as color and taste [70,71], and are well known for their antioxidant, anti-inflammatory, and antineoplastic properties [72,73,74]. In recent years, some of these compounds have been studied in combination with DOX, with the aim of protecting against the side effects produced by this drug at the brain level, where DOX provokes direct and indirect changes, producing the pathologic condition of chemobrain, where oxidative stress and inflammation play crucial pathogenic roles. The brain-protective activity of PheCs has also been extensively studied, and both indirect peripheral (related to their capacity to increase the blood flow in the cerebrovascular district) and direct actions at brain levels have also been suggested for them [75]. In particular, it has been reported that these natural compounds can exert their powerful antioxidant and ROS-scavenging effect at the brain level, where innate antioxidant defenses are limited, and a variety of drugs are not delivered at sufficient levels to be therapeutically active, with the BBB being a significant obstacle for drugs to reach the brain [76,77,78]. Moreover, the direct PheC activity inside the brain was also related to the ability to modulate specific receptors, neurotrophins, and signaling pathways [75]. Interestingly, some of these compounds were found to be able to cross the blood–brain barrier (BBB). Thus, they are also considered particularly suitable to exert all their healthy effects in this body region usually reached with difficulty by drugs [79,80,81,82]. Interestingly, once inside our body, through their digestion and extensive conjugation at the liver and colon levels, these compounds give rise to a variety of metabolites [83]. These metabolites, obtained in vivo and found in the circulation, have been suggested to have differential capacities for BBB transport in relation to their specific chemical structure and characteristics of lipophilicity [84]. In fact, by using an in vitro model mimicking the BBB environment, it was revealed that the potential of permeation of different PheC metabolites was consistent with their lipophilicity, and that the less polar methylated derivatives were more able to diffuse through membranes compared to the more polar PheC metabolites, such as sulfates and glucoronated compounds [85,86].

Here, we have considered the studies focused on this topic, restricting our analysis to those published in the last eight years (2016–2024). 

**Figure 3:**
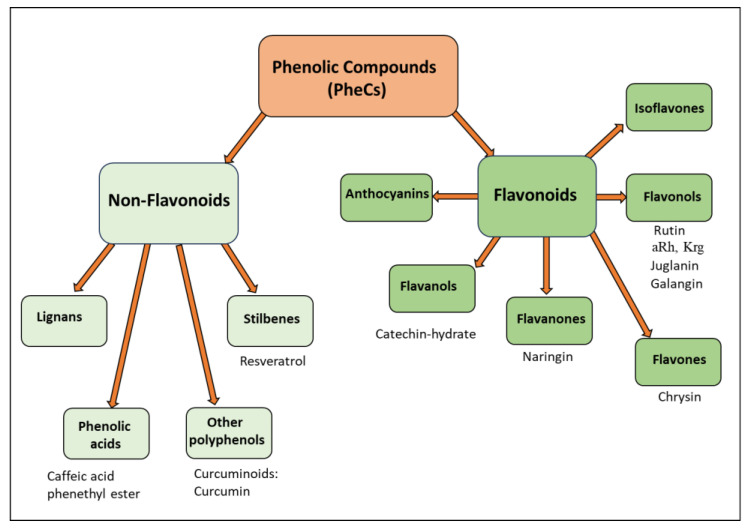
Classes of natural phenolic compounds (PheCs; classification based on the Phenol-Explorer database regarding the polyphenol content in foods, http://phenol-explorer.eu/compounds (accessed on 18 March 2024), version 3.6). The PheCs that have been evaluated in combination with doxorubicin for reducing chemobrain in the last 8 years (2016–2024) are indicated under the green boxes. aRh (α-Rhamnoisorobin) and Krg (Kaempferitrin) are glycosylated derivatives of the Flavonol Kaempferol.

PheCs include a large number of subclasses characterized by some peculiar differences in their structures that influence their biological properties. To describe these subclasses, we will use the same classification that was recently followed in a recent review of ours [87] focusing on dietary PheCs included in nanoparticles for cancer therapy. According to this classification, PheCs may be divided into two large subclasses: Flavonoids and Non-Flavonoids [Figure 3]. In turn, the Flavonoid subclass includes the Anthocyanins, Flavanols, Flavanones, Flavones, Flavonols, and Isoflavones, while the Non-Flavonoids subclass includes the Lignans, Phenolic Acids, Stilbenes, and a last subgroup including all the remaining and named “Other polyphenols”. Figure 3 reports the PheCs that have been investigated in preclinical models of chemobrain in the years 2016–2024 to establish their protective potential for possible future clinical applications. 

### 3.1. Effects of PheCs on DOX-Induced Chemobrain

To better analyze the results obtained in the studies, we have summarized them in four tables (Table 1, Table 2, Table 3 and Table 4), where we have reported the PheCs divided into classes, since the different classes may have unique characteristics and modulate many biological activities in a specific way.

The tables were organized to be self-explanatory, and, thus, we will not repeat the effects observed by the authors of each study in the text, but we will discuss the peculiarity of the outcomes, sometimes stressing the values or limitations of the studies. In the period 2016–2024, we identified twelve reports focusing on protective effects against DOX-induced chemobrain. These reports used either purified forms of PheCs (10 reports; see Table 1, Table 2 and Table 3) or plant extracts that, when examined for their content by the authors, were particularly rich in PheCs (2 reports, see Table 4). Eleven of these studies were performed by using rodent models, and among them, some included also in vitro or in silico studies, in order to provide more support to the results obtained in the in vivo models and to better identify the mechanisms implicated in the protective effect of the PheCs. Just one was performed only in vitro [88].

Altogether, the outcomes of all the studies analyzed agree and concur in demonstrating the potential of PheCs in reverting the deleterious effects observed after the treatment with DOX on the central nervous system, evaluated by behavioral tests and histologic examinations of the two brain locations mainly affected during the development and progression of the chemobrain condition (the HIP and frontal cortex). There is also agreement on the main biological and molecular mechanisms hypothesized to underlie the beneficial effects of the PheCs against DOX-induced chemobrain. These mechanisms are often related to the inhibition of the DOX-driven induction of oxidative stress and inflammatory process, as well as of the apoptotic pathways of cell death. In some cases, the ability of PheCs to revert the DOX-driven promotion of both endoplasmic reticulum (ER) stress or autophagy has also been involved, as well as their capacity to normalize the DOX-induced reduction in the production of neurotrophic factors and in neuronal growth.

There is only one study that was carried out exclusively in vitro [88], where the authors did not find any beneficial effect against DOX-induced cytotoxicity in neuronal cells by using two glycosides of the Flavonol Kaempferol, even though, in the same study, the same compounds were able to revert the damage induced in the cells by other cytotoxic and oxidant compounds, such as 6-hydroxydopamine or H_2_O_2_. 

Table 1(A,B) include the subclasses of Flavonoids, with Table 1(A) reporting the four studies performed using PheCs belonging exclusively to the class of the Flavonols (Rutin, Kaempferol, Galangin and Juglanin) [53,88,89,90] and Table 1(B) reporting three studies focusing on the effects of Naringin, which is a Flavanone [91], Catechin hydrate, which is a Flavanol [92], and Chrysin, belonging to the class of the Flavones [67]. The larger number of studies focused on the effect of different kinds of Flavonoids on chemobrain compared to other classes of PheCs is related to the fact that this is the largest class, containing about 6000 different substances that naturally and abundantly occur in a variety of plants [93]. Instead, as we look at the studies on chemobrain published on Non-Flavonoids, we observe that the studies analyzed by us regard only three different PheCs. In fact, the first study (Table 2) is focused on the effect of a Phenolic Acid (caffeic acid) [94], three studies on the effect of the Curcuminoid Curcumin, administered either as a free compound [95,96] or included in a nanoformulation designed for its better delivery [97] (Table 2), while the last three studies (Table 3) regard the effect of the Stilbene Resveratrol [95,98] or, in one case, one metabolic derivative of Resveratrol [99].

**Table 1:** (**A**). PheC (Flavonoids: Flavonols) modulation of anthracycline-induced chemobrain (2016–2024). (**B**) PheC (Flavonoids: Flavanols, Flavanones, and Flavones) modulation of anthracycline-induced chemobrain (2016–2024).

Anthracycline-Induced Chemobrain Model	Anthracyclin Administered	PheC Administered	Protective Effect(s) of the Combination with Respect to the Neurotoxic Effect of DOX Alone	Molecular Factors/Mechanisms Involved in the Protective Effects of PheCs against DOX-Induced Effects	Ref.
(**A**)					
In vitro: human neuroblastoma (IMR32) cells. In vivo: twelve-week-old, healthy female Wistar rats, weighing 180–230 g.	In vitro: 1 μM DOX In vivo: ten cycles with i.p. injection of DOX: 2.5 mg/kg, every 5 days for 50 days.	Rutin (RUT, Flavonol glucoside) In vitro: 100 μM RUT prior to DOX In vivo: RUT: 50 mg/kg (82 μmol/kg), starting one week before first DOX cycle and continuing for further 50 days, per os	In vivo: ↓ Impairment of episodic memory measured by ORT In vitro: ↓ DOX-induced IMR32 cell death, apoptosis, and intracellular ROS generation Restored neurite growth in differentiated IMR32 cells	In vitro: ↓ Cell death (apoptosis) ↓ Intracellular ROS generation ↑ Neurite growth In vivo: In the HIP and frontal cortex: ↓ TNF-α levels ↑ CAT, GSH, total thiols, and SOD levels	[53]
In vitro: undifferentiated and retinoic acid-differentiated SH-SY5Y neuroblastoma cells	DOX (0.375 and 0.5 μM for UN- and RA-SH-SY5Y, respectively).	Isoquercetin (5–50 μM) used as a reference compound. Kaempferol precursors (Flavonol glycosides) extracted from the leaves of *Maesa membranacea* (5–50 μM): α-Rhamnoisorobin (Kaempferol 7-O-α-rhamnoside, aRh) and Kaempferitrin (Kaempferol 3,7-di-O-rhamnoside, Krg)	In vitro: No protection by any Flavonoid against DOX-induced cytotoxicity in both UN-SH-SY5Y and RA-SH-SY5Y cells (measured as cell viability modification by WST-1 assay)	Not applicable	[88]
In vivo: adult male (8–10 weeks) Wistar rats (180 ± 25 g)	DOX (2 mg/kg, once/week for 4 weeks, i.p.)	Galangin, 50 mg/kg (185 µmol/kg), 5 times/week, for 4 weeks, per os	↓ Hippocampal neurodegeneration Cognitive and behavioral functions improved HIP antioxidant status ameliorated ↓ HIP DOX-induced inflammation reduced ↓ HIP long-standing and deleterious DOX-induced astrocyte activation	In the HIP: ↑ BDNF expression; ↓ Levels of oxidative indexes (MDA, NO, NOX-1) ↑ NRF2 and HO-1 expression; ↑ GSH tissue level ↓ Expression of inflammatory biomarkers: NF-κB p65, iNOS, TNF-α, IL-6, and IL-1β Modified expression of markers of astrocyte activation: ↑ GFAP expression and ↓ BDNF expression ↓ Expression of necroptosis markers: p-RIPK1, p-RIPK3, and p-MLKL	[89]
In vivo: six-week-old male Sprague Dawley rats: 180 ± 40 g	5 mg/kg DOX i.p. administered once a week for 3 weeks (total dose of 15 mg/kg).	Juglanin, 30 mg/kg/day (71 μmol/kg/day) for 4 weeks. For the first week, alone; for the remaining 3 weeks, in combination with the weekly DOX injection, per os.	↓ DOX-induced increase in immobility time (DOX-induced sign of anxiety) ↑ DOX-induced decrease in swimming and climbing time (DOX-induced depression-like behaviors). Improved learning and memory (measured by the Y-maze test, the Y-maze test, and the MWM test). ↓ DOX-induced neuroinflammation and oxidative stress. ↓ Histopathologic alterations (pyknosis, congested blood vessels, degenerated and swollen neurons)	Measured brain homogenates: ↓ MDA level ↑ SOD, CAT, and GSH levels ↓ AChE levels TNF-α, IL-1β, IL-6 and NF-κB content ↓ Caspase 3 activity	[90]
(**B**)					
In vivo: healthy male Wistar rats (150–200 g)	DOX (15 mg/kg, i.p.) administered on the 10th day of the Naringin treatment.	Naringin (Flavanone) 50 and 100 mg/kg (86 and 172 μmol/kg) for 14 days, i.p. injected	Alleviation of anxiety-like behavior (time spent in open arms, closed arms) and depressive-like behavior (immobility time, swimming time)	In vivo: In the HIP: ↓ plasma corticosterone, TNF-α and IL-1β levels ↑ mitochondrial complexes I, II activities and mitochondrial redox activity ↑ levels of serotonin and dopamine	[91]
In vitro: undifferentiated and RA-differentiated neuroblastoma IMR-32 cells In vivo: twelve-week-old healthy male rats weighing 200–230 g	In vitro: 1 and 2 μg/mL DOX In vivo: 2.5 mg/kg b.w. i.p. injection (10 cycles every 5 days)	Catechin hydrate (Flavanol) In vitro: 31–250 μg/mL In vivo: 100 mg/kg b.w. (324 μmol/kg) for 57 days including one week prior to the first cycle of DOX, per os	In vitro: ↑ undifferentiated cell viability ↓ degeneration, ↑ neurite length and prevention of DOX-induced cell cycle arrest in differentiated cells In vivo: prevention of DOX-induced memory deficit by the NORT assay	In vivo: ↓ oxidative stress, acetylcholine esterase and neuroinflammation (reduced nitrite and MPO levels) in the HIP and cerebral cortex	[92]
In vivo: eight-week-old male Sprague Dawley rats 180–200 g	DOX, 2 mg/kg/week, i.p. for 4 weeks.	Free Chrysin (Flavone), 30 mg/kg (118 μmol/kg), per os or Chrysin formulations (transfersomal and chitosan composite vesicles), 0.5 mg/kg, 5 times/week/4 weeks intranasal delivery	↓ histological changes and neurodegeneration ↑ cholinergic transmission ↑ memory acquisition and spatial memory (y maze test and Moris water maze test)	In HIP and PFC: ↑ GSH levels and CAT activity ↓ level of hydrogen peroxide and lipid peroxidation Inhibition of TLR4/NF-κB/NLRP3 signaling pathway ↓ caspase-1 and IL-1β protein expression ↓ AchE enzyme	[67]

**Table 2:** PheC (Non-Flavonoids: Phenolic Acid and Curcuminoids) modulation of anthracycline-induced chemobrain.

Anthracycline-Induced Chemobrain Model	Anthracycline Administered	PheC Administered	Protective Effect(s) of the Combination with Respect to the Neurotoxic Effect of DOX Alone	Molecular Factors/Mechanisms Involved in the Protective Effects of PheCs against DOX-Induced Effects	Ref.
Sprague Dawley male rats (200–250 g)	DOX (2 mg/kg, once a week, i.p. for 4 weeks).	CAPE, 10 or 20 μmol/kg/day, 5 days per week, for 4 weeks, i.p. injected.	Counteraction of spatial learning and memory impairment (measured by MWM test; passive avoidance test; assessment of locomotion) ↓ Hippocampal and PFC neurodegenerative changes (nuclear pyknosis and degeneration of the neuronal cells)	In the HIP and cortex: ↓ levels of inflammatory biomarkers (GFAP, COX-2, NF-κB p65, TNF-α) Normalization of GSH and MDA levels ↑ ACh levels ↓ Active caspase-3 levels	[94]
Male adult Wistar rats (160 to 180 g)	A single dose of DOX (20 mg/kg, i.p.) on the 10th day	Nanocurcumin, 50 mg/kg/day, for 9 days (before the DOX injection) and for further 4 days, per os	Not determined	Normalization of oxidative stress parameters: in the cortex: (↓ MDA and NO levels) in the HIP: (↑ GSH levels) in the cortex and HIP: no effect on the DOX-induced inhibition of AchE and MAO activities normalization of DOX-induced ↑ levels of dopamine	[97]
Male Wistar rats, 3 months of age (290 ± 20 g)	DOX, a weekly dose of 2.5 mg/kg for 4 weeks, i.p.	Curcumin, 10 mg/kg/day (27 μmol//kg/day) for 28 days, started on the same day as the first DOX injection, per os	↓ Short- and long-term memory impairment (NORT test at 3 and 24 h after habituation)	In frontal cortex, hypothalamus and HIP: ↓ GFAP (in astrocytes) and Iba1 (in microglia) expression (markers of strong microglial and astrocyte response, due to the neuroinflammatory response)	[95]
Male Sprague Dawley rats (200–220 g)	DOX (2.5 mg/kg injected i.p. every 2 days)	Curcumin, 30 mg/kg/day (81 μmol/kg/day) for 3 weeks starting 1 week before DOX administration, per os	Improvement of DOX-induced symptoms of depression: (↓ sucrose preference in SPT, ↑ latency time in NSFT, immobility time in FST and number of crossings in OFT ↓ histopathological changes (nuclear pyknosis) and apoptosis (measured by Tunel test)	In the HIP: ↓ oxidative stress biomarkers (↓ HNE-positive cells, ↓ levels of MDA and NO, ↓ CAT and GPx activities ↓ Endoplasmic reticulum (ER) stress biomarkers (↓ CHOP and GRP78 expression) Modulation of autophagy biomarkers (↓ LC3-II/LC3-I ratio, ↓ Atg-5, Atg-7, and Becn1, expression; ↑ p62 expression) Activation of NRF2-ARE pathway (↑ NRF2 and Keap-1 nuclear expression; ↑ NQO-1 and HO-1 expression)	[96]

**Table 3:** PheC (Non-Flavonoids: Stilbenes) modulation of anthracycline-induced chemobrain (2016–2024).

Anthracycline-Induced Chemobrain Model	Anthracycline Administered	PheC Administered	Protective Effect(s) of the Combination with Respect to the Neurotoxic Effect of DOX Alone	Molecular Factors/Mechanisms Involved in the Protective Effects of PheCs against DOX-Induced Effects	Ref.
Female C57/BL6J mice (18–20 g)	DOX in combination with other chemotherapies: DTX + DOX + CP, 10/10/40 mg/kg (DAC) Three i.p. injections at 2-day intervals	Resveratrol, 50 and 100 mg/kg/day (219 or 438 μmol/kg/day) for three weeks, beginning one week before the DAC treatment per os	↓ Anxiety levels and locomotor activity (open-field test) ↑ Cognitive performance (by Morris water maze test) ↑ PFC and hippocampal neuronal activity (by MEMRI test)	In serum, whole brain, PFC, and HIP: ↓ TNF-α and IL-6 levels ↑ IL-4 and IL-10 levels In PFC and HIP: ↑ Neuroplasticity biomarker expression (BDNF, TrkB, amino acid neurotransmitter receptors, and CaMKII) Regulation of components of PPARγ/NF-κB signaling (↑ PPARγ expression, ↓ p-p65, and p-IκBα expression)	[98]
Forty male Wistar rats, 3 months of age and weighing 290 ± 20 g	DOX, 2.5 mg/kg/week for 4 weeks, i.p.	Resveratrol, 10 mg/kg/day (44 μmol/kg/day) for 28 days, started on the same day as the first DOX injection, per os	↑ Long-term memory impairment (NORT at 24 h after habituation).	In frontal cortex, hypothalamus and HIP: ↓ GFAP (in astrocytes) and Iba1 (in microglia) expression (markers of strong microglial and astrocyte response, due to the neuroinflammatory response)	[95]
Six-week-old male Sprague Dawley rats (210–230 g)	DOX, 2 mg/kg, once a week for 4 weeks, i.p.	Polydatin, 50 mg/kg/day (128 μmol/kg/day) for 4 weeks, per os	↑ Spatial learning and memory ability in rats (Morris water-maze task). Inhibition of nuclear pyknosis and degeneration of neuronal cells in the HIP	In HIP: ↓ Oxidative stress (↓ MDA levels, ↑ GSH levels; ↓ NRF2 expression) ↓ Inflammation (↓ TNF-α, PGE-2 and COX-2 levels; ↓ p-IκB and p-p65 expression) ↓ Apoptosis (↓ cleaved caspase-3 and -9 expression)	[99]

#### 3.1.1. Flavonoids and DOX-Induced Chemobrain

**Figure 4:**
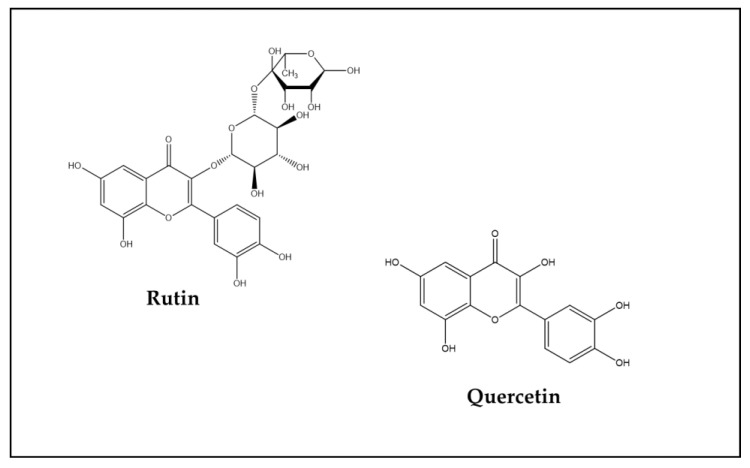
Chemical structure of Rutin and Quercetin.

In the first study carried out with Flavonoids [53], Rutin, a Flavonol which is also known by the names of Rutoside or vitamin P and which is widely distributed in vegetables and fruits, was used [100]. The Rutin moiety is a glycosylated derivative of the Flavonol Quercetin, which is, in this case, bound to the disaccharide rutinose [101,102] (Figure 4).

**Figure 5:**
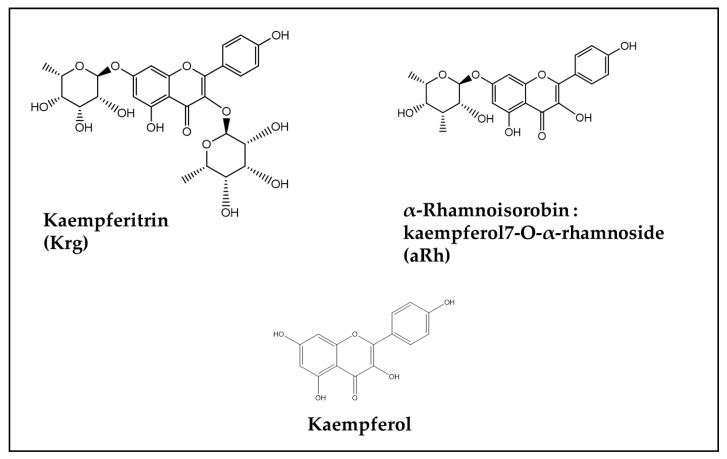
Chemical structure of Kaempferol and its derivatives Kaempferitrin (Kaempferol 3,7-di-O-rhamnoside, Krg) and α-Rhamnoisorobin (kaempferol7-O-α-rhamnoside, aRh).

In this rutinose-bound form, besides showing the antioxidant and anti-inflammatory effects common to all the other Flavonoids, this bioactive compound has also been reported to exert powerful anticancer and antimicrobial effects [103,104]. In Table 1(A), it is shown that all the main mechanisms of DOX-induced damage or death of cells at the brain level (apoptosis, oxidative stress growth of neurites, inflammation) were affected by the beneficial activity of Rutin. Similar healthy properties are reported to be shared by another Flavonol, Kaempferol [105,106,107,108], which, compared to the best-known Flavonoid Quercetin, has one additional OH group on one of the aromatic rings of its molecule (compare Figure 4 and Figure 5).

Due to its powerful bioactivity, the application of Kaempferol was recently hypothesized to be useful in a series of diseases pathogenically related to inflammation, such as some cardiovascular diseases, diabetes mellitus, and asthma, and in disorders related to microbial contamination [109]. Similarly to Quercetin, found in many plants and known for its healthy and pleiotropic bioactivities [110], Kaempferol is also found easily glycosylated in nature. However, as already reported above, Jantas et al. [88] found that two glycosylated derivatives of Kaempferol, i.e., α-Rhamnoisorobin (Kaempferol 7-O-α-rhamnoside, aRh) and Kaempferitrin (Kaempferol 3,7-di-O-rhamnoside, Krg), did not succeed in reverting the cytotoxic effect of DOX in neuroblastoma cells studied in vitro (Figure 5) (Table 1(A)). However, it could be possible that the lack of effects in this in vitro study could be simply related to the use of glycosylated derivatives, since it is known that the free phenolic forms represent the active forms, and glycosylases are important to transform the glycosylated precursors to the active forms [111]. However, these negative results do not preclude that Kaempferol or its glycosylated derivatives could induce the same beneficial effects observed with the other Flavonols in vivo, especially since Juglanin, which is a Flavonol constituted by Kaempferol glycosylated with the pentose L-Arabinofuranose and known to exert powerful anti-inflammatory, antioxidant, and anticancer activities [112,113,114], was also among the other Flavonols recently reported to exert powerful neuroprotection against DOX-induced chemobrain in vivo [90] (Table 1(A)). It is interesting to notice that this more recent finding is in agreement with a previous observation according to which Juglanin is able to prevent neuroinflammation in animal models of Parkinson’s disease [115]. Therefore, altogether, these findings suggest that further studies are needed to evaluate whether the two Kaempferol glycosides aRh and Krg, so far tested only in vitro against DOX-induced neurotoxicity, could exert protective effects in in vivo models. 

Galangin is the last among the Flavonols to be tested against DOX-induced chemobrain [89] (Table 1(A)). In nature, it can be found particularly concentrated in propolis and in *Alpinia officinarum*, a plant commonly known as galanga, after which it has been named. It shows pleiotropic bioactivities, having been demonstrated that is not only able to inhibit oxidative stress and inflammation but also exert antifibrotic, antimicrobial, and antihypertensive effects and even reduce vascular abnormalities in rat models of metabolic syndrome [116,117]. For some of these properties, its possible application in cancer was hypothesized, as well as in a series of inflammatory and degenerative diseases, including skin diseases, rheumatoid arthritis, osteoarthritis, neurodegenerative diseases, and osteoporosis [116,118,119,120]. In particular, Abd El-Aal et al. [89] focused on Galangin’s effect on chemobrain (Table 1(A)) since previous results had proven its ability to attenuate oxidative stress and inflammation in animal models of pathologies where these processes play a main pathogenetic role, such as psoriasis, hepatitis, osteoarthritis, or colitis [121,122,123,124]. The results obtained confirmed (Table 1(B)) that the beneficial effects of this compound against DOX-induced brain degeneration were related to its capacity to reduce brain oxidative stress, inflammation, and degeneration, as shown by the increased HIP expression of the cell repair/growth index, BDNF. Moreover, it also reduced DOX-induced necroptosis, as indicated by the decreased expression of some of the factors involved in this mode of cell death (p-RIPK-1, p-RIPK-3, and p-MLKL) in the HIP.

The first Flavonoid included in Table 1(B) (reporting results obtained with Flavonoids that do not belong to the Flavonol class) is the Flavanone Naringin. The authors of [91] related its protective effect against DOX-induced chemobrain to both its anti-inflammatory and antioxidant activities. However, similarly to what was observed above in a more recent study carried out with the Flavonol Galangin [89], the beneficial effect of Naringin could also be related to the neurotrophic activity that this bioactive compound is able to exert. This finding is also in agreement with what was recently observed by Yilmaz et al. [125] in an ischemia–reperfusion animal model, which showed that Naringin was able to revert impaired neurogenesis to normal and increase the levels of BDFN in the HIP and frontal cortex of the animals. 

In the second study of Table 1(B) [92], the authors administered Catechin hydrate to rats treated with DOX and confirmed the antioxidant and anti-inflammatory ability of this Flavanol in the animal model of DOX-induced neurotoxicity that they used. In fact, this compound, found at high levels in green tea and other plants [126], similar to the most famous and well-studied Flavanol epigallocatechin-3-gallate (EGCG), is considered a powerful antioxidant that also shows anti-inflammatory activities. Moreover, it is worthy of note that the powerful anticancer and antifibrotic activities of this compound have been demonstrated [127,128], which make its potential use along with other antineoplastic agents particularly interesting.

The last Flavonoid that has been evaluated for its ability to reduce the effect of DOX on brain functions [67] (Table 1(B)) is the Flavone Chrysin, which appears particularly appropriate for this role, since it is not only known for its antioxidant and anti-inflammatory ability [129,130] but also for its capacity to modulate neurotrophic factors and the activities of neurotransmitters in the central nervous system [131]. The Ibrahim et al. study [67] appears to be particularly interesting since Chrysin was administered to the DOX-treated rats either in the native form per os or enclosed in transferosomal lipid vesicles and chitosan composite vesicles designed by the authors for a more direct nose-to brain delivery. In fact, transferosomes are constituted by a lipid bilayer incorporating single-chain surfactants that make them particularly flexible and apt to cross biological membranes [132], while chitosan bound to liposomal vesicles enhances their residence time at the level of the nasal mucosa, thus improving their brain uptake [133]. Since it was previously observed that high oral doses (100 mg/kg) of Chrysin were needed to obtain appreciable beneficial effects in neurodegenerative disorders [134], the authors evaluated whether low doses of Chrysin (0.5 mg/kg) embedded in the two newly designed nanoformulations could match or even improve the therapeutic efficacy of higher doses of free Chrysin (30 mg/kg) in a rat model of chemobrain. They observed that, independently from the route of administration or type of formulation, Chrysin reduced DOX-induced cognitive impairment and the histological alterations and neurodegeneration induced by DOX. However, its therapeutic potential was markedly enhanced when it was intranasally administrated inside both the nanovesicles. Moreover, the sixty times lower doses of Chrysin delivered as nanoformulations were sufficient to obtain comparable or even higher beneficial effects, with respect to those observed with the higher oral dose, on DOX-induced chemobrain. In particular, the authors observed that the superior efficiency of the Chrysin nanoformulations in reverting the DOX-induced memory impairment was related to their enhanced ability to inhibit acetylcholinesterase, oxidative stress, and TLR4-NF-kB-NLRP3 inflammasome pathway.

#### 3.1.2. Non-Flavonoids and DOX-Induced Chemobrain

Instead, among the Non-Flavonoids tested against DOX-induced chemobrain and reported in Table 2, we found the ester of caffeic acid and phenethyl alcohol (caffeic acid phenethyl ester, CAPE) [94], which is the form of caffeic acid naturally present at high levels in honeybee propolis, largely used in traditional medicine [135,136]. CAPE has been demonstrated to be a powerful natural antioxidant and anti-inflammatory agent. Moreover, it has been reported to exert potent immunomodulatory, anti-viral, and antibacterial activities and to act as a promoter of wound healing and a potential antineoplastic agent [137]. As shown in Table 2, Ali et al. [94] observed that the protective effect of this compound was related to its ability to inhibit oxidative stress and the inflammatory process in the brain regions critically related to DOX-induced chemobrain development (the HIP and PFC). Of interest, they observed that CAPE reverted the tissue level of acetylcholine (ACh) to normality after it had been dramatically reduced by the DOX treatment. This DOX-induced alteration in ACh levels was related by the authors to the previously observed DOX-induced oxidative stress and the ROS-induced activation of the acetylcholinesterase enzyme [56,138]. Thus, the action of CAPE on Ach was interpreted as further important evidence that the antioxidant activity of this compound was involved in the protective effect observed against the neurotoxicity and altered spatial learning and memory deficits induced by DOX. In agreement with this, previous reports have demonstrated that acetylcholinesterase inhibitors protected against DOX-induced behaviors and symptomatology of DOX-induced chemobrain [139]. 

Curcumin is the second Non-Flavonoid that was evaluated for its activity against DOX-associated chemobrain in three different studies (Table 2). It deserves particular attention since, among the PheCs, it is one of the most studied due to its pleiotropic beneficial effects on health, being able to powerfully function as an antioxidant, anti-inflammatory, antimicrobial, antithrombotic, and antineoplastic factor [140,141]. It is the main bioactive ingredient of the tropical plant *Curcuma longa*, which has been used in Ayurveda and Chinese traditional medicine for thousands of years for its medicinal properties [140,142]. Moreover, the spices derived from the root of this plant and containing high levels of Curcumin, even though originally used only in the Eastern countries, have now extended its culinary usage everywhere. Due to the large spectrum of its bioactivities, Curcumin has been considered as a possible valid support for improving patient conditions in various diseases, including diabetes mellitus, Non-Alcoholic Fatty Liver Disease, cancer, COVID-19, autoimmune diseases, and psychological disorders [143,144,145,146,147]. However, it is becoming increasingly clear that Curcumin’s therapeutic applications are quite limited due to its poor aqueous solubility, absorbance, and bioavailability, as well as its pharmacokinetic profile. Thus, we are now witnessing an upsurge in the research exploring the possibility of developing new formulations containing Curcumin, especially by encapsulating it in nanoparticles able to improve its absorption, specific delivery, and bioavailability, and, thus, its therapeutic effects [148]. Interestingly, one of the Curcumin studies examined was conducted by using a nanoformulation encapsulating it (NanoCUR) [97]. However, in this study, the effects observed were not compared to those obtained with a control represented by free Curcumin. Moreover, the composition of the nanoformulation and its chemical–physical characteristics were not reported since the authors used a commercial Nanocurcumin product (from the company One Planet Nutrition). This means that demonstrating the possible increased bioactivity of Curcumin when included in nanoparticles was not a main objective of the authors. Moreover, the authors failed to demonstrate the effective presence of DOX-induced chemobrain and related neurotoxicity in the DOX-treated animals (by evaluating altered behaviors and/or objective histological modifications induced by DOX), as well as the possible reversion of these phenomena by nanoCUR. Instead, they reported the nanoCUR-driven normalization of some DOX-induced alterations in biochemical parameters of oxidative stress, as well as in the levels of the neurotransmitter dopamine, both measured in the brain districts (the HIP and frontal cortex) usually analyzed in these kinds of studies. On the other hand, it is worth noting that the other two studies carried out with free Curcumin [95,96] (Table 2), even though carried out with different experimental designs and dosages of both DOX and Curcumin, obtained interesting and complementary results on the protective effect of this PheC on inflammation [95], oxidative stress, ER stress, and autophagy [96], some of the main processes known to underlie the DOX-induced alteration in mnemonic functions and brain morphology. 

**Figure 6:**
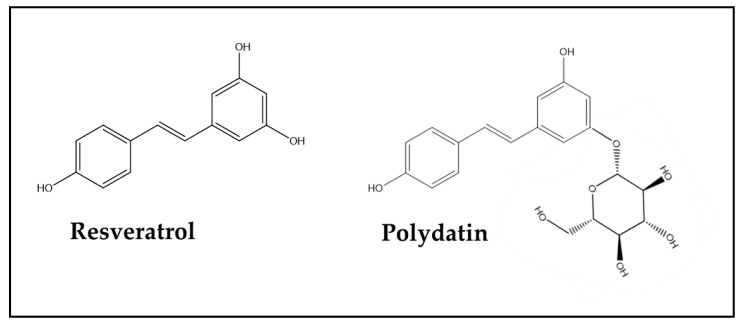
Chemical structure of Resveratrol (3,4′,5,-trihydroxy-trans-stilbene) and its precursor Polydatin (resveratrol-3-O-β-mono-D-glucoside).

Table 3 shows the results of three studies performed with Resveratrol, a member of the Stilbene group of the Non-Flavonoid class of PheCs, which is present in considerable amounts in grapes and wine, peanuts, nuts, soy, berries, and tea [149,150,151]. This bioactive natural product is well known for its strong antioxidant, anti-inflammatory, vasculoprotective, and anticancer properties, as well as for its immunomodulatory activities [152,153]. Due to these characteristics, Resveratrol’s possible therapeutic applications for the adjuvant therapy of a variety of chronic diseases, including diabetes mellitus type 1 and rheumatoid arthritis, have recently been suggested [154,155]. One of the studies considered here used Polydatin, which is a glucoside of Resveratrol, and probably the most abundant form of Resveratrol in nature [99] (Figure 6).

Polydatin is a metabolic precursor of Resveratrol that shows comparable efficacy to free Resveratrol. In fact, the results of the three studies concurred in demonstrating that, independently of its chemical form, Resveratrol showed similar effects in reverting DOX-induced chemobrain manifestations, as well as the level or activity of molecular factors and pathways involved in neuroinflammation. Moreover, Polydatin was also found to be able to reduce DOX-induced oxidative stress [99], which was not evaluated in the other two studies. Instead, in the study by Shi et al. [98], free Resveratrol was also found to be able to revert the neuroplasticity loss induced by the DOX treatment. This study [98] appears to be particularly valuable for its methodology, since the authors did not use DOX alone to induce chemobrain in the animals, but rather a combined treatment with Docetaxel (DTX)/DOX/CP, in order to better mimic one of the first-line treatments currently administered for the treatment of advanced BCa patients. In fact, patients treated for BCa and affected by chemobrain are the most numerous and studied, since their post-treatment life expectancy is increasing and allows this long-term complication to occur more easily than in patients treated for other tumors with lower life expectancies. Moreover, this and other combination therapies are now increasingly used, since they have some advantages over single-agent therapies, such as the possibility to reduce the dosage of each single drug and decrease the probability of tumor resistance development [156]. Moreover, these combinatory regimens allow the different and specific antineoplastic activities of multiple anticancer agents to be exerted simultaneously in the same patients. However, despite these advantages, it has been observed that these combinatory regimens still induce considerable side effects, including chemobrain [157]. Therefore, experimental models using combinations of drugs are particularly appropriate for studying the biological and molecular mechanisms underlying the side effects induced by these therapeutic regimens, as well as possible therapeutic strategies to inhibit the development of these adverse effects or attenuate them. Moreover, it is noteworthy that, in the same study [98], female animals were used, as DOX is more frequently used either as a single agent or included in a combinatory regimen for the cure of BCa, a typical female neoplasm.

Altogether, we notice that comparable beneficial effects were reported for all the different PheCs, both in reverting DOX-induced behavioral alterations and in reducing the morphological modifications at the brain level. These effects were obtained irrespective of the dosages, route, and timing of administration. In fact, even though most of the authors administered the PheCs per os (via gavage), Naringin [91] and CAPE [94] were i.p. injected, and Chrysin embedded in nanoparticles was intranasally delivered. Moreover, in most studies, the compounds were administered at a dose of 50 mg/kg b.wt., with some researchers administering lower doses (10 or 30/mg/kg b.wt.) or higher doses (100 mg/kg b.wt.). In order to better understand and compare the effect on a dose basis, we also reported them in the tables converted into µmol/kg/body weight (b.wt.). In this way, the administered doses varied from 10 µmol/kg of CAPE to 342 µmol/kg/b.wt. or 438 µmol/kg/b.wt. of Catechin hydrate and Resveratrol, respectively.

Moreover, sometimes, the PheC treatment was started 7-10 days in advance with respect to that with DOX, as in the case of Rutin [53], Juglanin [90], Naringin [91], Catechin hydrate [92], Nanocurcumin [97], Resveratrol [98], and Curcumin [95]. Interestingly, Curcumin was used in two different studies and administered in different ways. In fact, whereas, in the first study, 30 mg/kg/day (81 μmol/kg/day) Curcumin was administered one week before the beginning of DOX treatment [95], in the second one [96], 10 mg/kg/day (27 μmol/kg/day) Curcumin was administered simultaneously to DOX, and the effects on the DOX-induced memory impairments were perfectly comparable. This could suggest that preventive treatment with Curcumin is not needed to protect from the devastating effect of DOX on brain functions. However, the usefulness of preventive treatment with Curcumin should not be ruled out, since it can be observed that the treatment with the highest dose of Curcumin that was preventively administered in the first study was needed to contrast chemobrain-related effects obtained with a much higher dose of DOX (7.5 mg/kg/week). Instead, in the second study, the lower dose of Curcumin was sufficient, even though it was administered simultaneously to DOX, probably since the DOX dose used was much lower (2.5 mg/kg/week). Similarly, Resveratrol was used in two studies at different dosages, and both showed comparable abilities in reverting a series of brain dysfunctions typical of chemobrain. In the first study [98], Resveratrol was used at 50–100 mg/kg/day (219–438 μmol/kg/day) starting one week before DOX, whereas, in the second one [95], it was used at 10 mg/kg/day (44 μmol/kg/day) and given simultaneously to the DOX treatment. However, in this case, a comparison in terms of dosages used and timing of administration is also not possible, since, in the first study [98], a higher dose of Resveratrol was administered to contrast the effect of a combinatory regimen represented by 10 mg/kg DOX and two other chemobrain-inducing drugs, i.e., DTX (10 mg/kg) and CP (40 mg/kg) (three i.p. injections at 2-day intervals). On the other hand, in the second study [95], Resveratrol was given at a lower dosage to contrast the effect of 2.5 mg/kg DOX alone administered once a week for 4 weeks.

#### 3.1.3. Extracts of Plants with High Contents of PheCs and DOX-Induced Chemobrain

Finally, Table 4 reports two recent studies [158,159] carried out by administering extracts of the plants *Carissa macrocarpa* and *Thunbergia erecta*, both with high contents of PheCs. The peculiarity of these two studies is that the authors accurately performed the chemical characterization of the plant extracts, identifying the PheC constituents present in the prevalent polyphenolic fraction by using either liquid chromatography–electrospray ionization–tandem mass spectrometry (LC-ESI-MS/MS) or ultra-performance (UPLC)-ESI-MS/MS.

In any case, the design of the two studies is quite similar to the others already examined, and the authors first evaluated the brain-related side effects of the treatment with DOX, combined or not with a parallel treatment with PheCs. In particular, they evaluated the changes in the animal behaviors especially related to memory and, correspondingly, post-mortem, they examined the histology of HIP and PFC, since, as also demonstrated by all the other studies, the function of memory is strictly related to these two brain districts. Of interest, these two studies confirmed their results by using in silico experiments. In the first one [158], on the basis of the results of the molecular docking experiments of TNF-α-converting enzyme (TACE), the authors observed that some PheCs present at high levels in the *Carissa m*. extract [procyanidin B6 (a Catechin dimer), procyanidin B5 (epicatechin dimer), epicatechin3-o-β-D-glucopyranoside, and hyperoside (a galactoside of Quercetin)] showed the highest affinity for TACE and, thus, could be responsible for the inhibitory activity against DOX-induced neuroinflammation. In the second study [159], molecular docking experiments were performed within the active site of the Receptor for Advanced Glycation End-products (RAGE), since the authors had found that the extracts obtained from *Thunbergia erecta* interfered with the High-Mobility Group Box 1 (HMGB1)/RAGE pathway by inhibiting the expression of HMBG, RAGE, p65, and IL-1β. Among the polyphenolic compounds identified in the LC-ESI-MS/MS analysis, Rosmarinic acid (an ester of caffeic acid and 3,4-dihydroxy phenyl lactic acid) was found to have the highest affinity for the active site of RAGE and, thus, could be partially responsible for the protective activity of the *Thunbergia erecta* extracts against the RAGE-dependent induction of ROS production and inflammation implicated in DOX-induced chemobrain.

**Table 4:** PheC-rich plant extracts and modulation of anthracycline-induced chemobrain.

Anthracycline-Induced Chemobrain Model	Anthracycline Administered	Administered Sources of PheCs	Protective Effect(s) of the Combination with Respect to the Neurotoxic Effect of DOX Alone or in Combination with CP	Molecular Factors/Mechanisms Involved in the Protective Effects of PheCs against DOX-Induced Effects	Ref.
Male Wistar rats, weighing 120–160 g	DOX 2.5 mg/kg/week for 4 weeks, i.p.	*Carissa macrocarpa* leaves polar fraction of hydromethanolic extract containing high % of PheCs (evaluated by UPLC-ESI-MS/MS profiling) 100–500 mg/kg/day *Carissa m.* extract for 4 weeks, per os	↓ Short- and long-term memory impairments (measured by Y-maze test and NORT, respectively) in a dose-dependent manner Partial recovery from histopathological alterations in the HIP (↓ perivascular space; ↓ neuropil vacuolation; ↓ neuroglia cell pyknosis and hyperchromasia; ↓ pyramidal cell degeneration)	↓ ROS and TNF-α serum levels ↓ caspase-3 expression in the HIP ↑ NGF brain levels in a dose-dependent manner	[158]
Male Wistar rats (180–200 g, 8 weeks old)	DOX (4 mg/kg/week, i.v.) in combination with CP, (40 mg/kg/week, i.v.) in the rats’ tail vein once per week for 3 weeks	*Thunbergia erecta* leaf ethyl acetate fraction of alcohol extract (TEAF), (50, 100, or 200 mg/kg) containing 21 characterized phytoconstituents, mostly PheCs (especially Phenolic Acids and Flavonoid glycosides), 5 times per week for 3 weeks, per os	↓ Learning and memory impairments (measured by NORT, Morris water maze tests, and step-through passive avoidance test) In PFC and HIP: ↓ Histological alterations in a dose-dependent manner Normalization of oxidative stress markers (CAT activity; MDA, GSH, hydrogen peroxide level)	↓ Protein expression of inflammation-related factors (HMBG1, RAGE, p65 NF-κB, and IL-1β)	[159]

## 4. Conclusions and Future Perspectives

**Figure 7:**
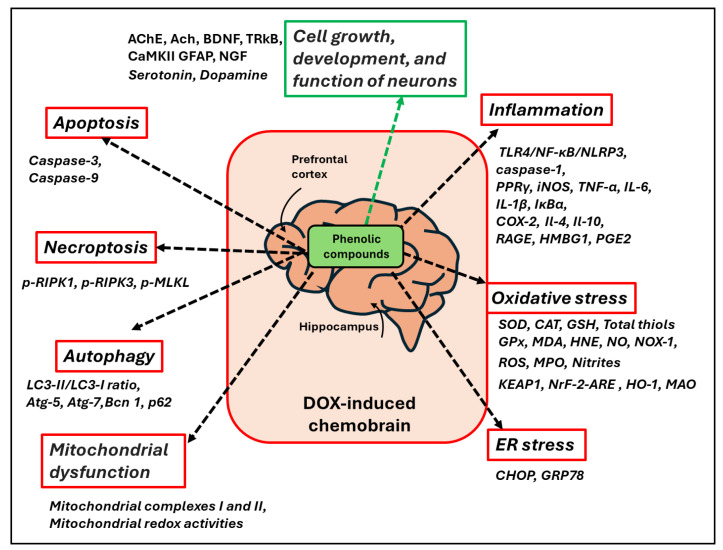
**Modulatory activities of PheCs on several processes implicated in the development and progression of DOX-induced chemobrain.** Black dashed arrows: modulation of processes indicated (in red boxes); for each process, molecular factors/pathways modulated by PheCs in the brain are indicated in italics. Green arrow: normalization of some physiological processes (in the green box) affected by DOX treatment in the brain; molecular factors modulated by PheCs.

All the studies analyzed concur in demonstrating the powerful potential of PheCs against the development of DOX-induced chemobrain and suggest that the main mechanisms involved are related to their capacity to reduce oxidative stress and inflammation induced by DOX, as well as to reestablish more physiological levels of neurotrophic factors and neurotransmitters (Figure 7). The figure also shows other processes implicated in DOX-induced chemobrain (ER stress, autophagy, mitochondrial dysfunction, apoptosis, and necroptosis) that have been demonstrated to be modulated by PheCs.

It is worth noting that only two out of the twelve studies analyzed here used preclinical models that better mimicked the conditions of most patients affected by chemobrain, i.e., female patients affected by BCa and therapeutically treated not with a single-drug therapy, but with a combinatory regimen. In fact, only Ramalingayya et al. [53] and Shi et al. [98] used female animals, while only El-Din et al. [159] and Shi et al. [98] treated the animals with DOX/CP or DOX/CP/DTX, respectively. Therefore, for future studies, it would be more appropriate to use similar conditions that better mimic those actually found in clinical practice. Moreover, it should be underlined that only in one [67] out of the twelve studies analyzed by us was there an attempt to verify whether the inclusion of these PheCs in innovative delivery nanosystems could enhance their therapeutic potential against the development of neurotoxicity and chemobrain. This strategy has recently been widely used for obtaining more efficient and specific delivery of drugs or natural bioactive compounds, including PheCs [87,160,161,162,163]. In particular, we have recently developed different delivery systems for natural products known for their anti-inflammatory and antineoplastic effects [164,165,166,167]. In some cases, this strategy was finalized to reduce the amounts of the antineoplastic drug administered and obtain antineoplastic effects comparable to those of larger amounts [160]. Moreover, in a recently published review of ours, we analyzed the studies where PheCs were used as the main components of a variety of nanosystems designed to carry anticancer drugs more efficiently and specifically to tumors [87]. On this basis, we suggest that these kinds of innovative nanostrategies could be used in two ways in studies focusing on the secondary neurotoxic effects induced by antineoplastic treatment with DOX. On the one hand, DOX could be administered enclosed in one of the multiple nanoparticles recently developed and specifically targeted for different types of cancer [168,169,170], particularly for BCa [28,171]. This strategy would allow the delivery of this drug more specifically to the tumor and impede it from reaching the brain and other non-specific districts in high proportions. Moreover, it would allow the use of lower doses of DOX, which are associated with fewer side effects. On the other hand, PheCs could be simultaneously administered enclosed in different nanoparticles designed for the specific and increased delivery of these compounds to the nervous central system, with the aim of acting directly there to avoid the development of DOX-induced chemobrain. Of note, the nose-to-brain delivery suggested by Ibrahim et al. [67] for Chrysin seems to be a valuable approach to obtain a stronger effect with lower doses of PheCs administered for reducing DOX-induced chemobrain. This treatment could precede treatment with DOX and continue for long periods after the anticancer treatment is over, given the safety profile of PheCs, with the aim of obtaining long-lasting preventive and curative effects on DOX-induced neurotoxicity.

## Data Availability

Not applicable.

## Abbreviations

Ach	acetylcholine
AchE	acetylcholinesterase
ARE	antioxidant response element
ATG	autophagy-related protein
BDNF	brain-derived neurotropic factor
Becn1	beclin 1
CaMKII	calmodulin-dependent protein kinase II
CAPE	caffeic acid phenethyl ester
CAT	catalase
CHOP	C/EBP homologous protein
COX	cyclooxygenase
CP	cyclophosphamide
CUR	Curcumin
DTX	docetaxel
FST	forced swimming test
GFAP	anti-glial fibrillary acidic protein
GPx	glutathione peroxidase
GRP78	glucose-regulated protein 78
GSH	reduced glutathione
HIP	hippocampus
HMBG1	High-Mobility Group Box 1 protein
HNE	4-hydroxynonenal
HO-1	heme oxygenase-1
Iba1	ionized calcium-binding adaptor molecule 1
IκB	inhibitor of the nuclear factor κ
iNOS	inducible nitric oxide synthase
MAO	monoamine oxidase
MDA	malondialdehyde
MEMRI	manganese-enhanced magnetic resonance imaging
MLKL	mixed-lineage kinase domain-like protein
MPO	mieloperoxidase
NF-κB	nuclear factor κB
NGF	nerve growth factor
NLRP3	Nod-like receptor pyrin-containing 3
NO	nitric oxide
NORT	novel object recognition test
NOX	NADPH oxidase-1
NQO-1	NAD(P)H quinone oxidoreductase
NRF2	nuclear factor (erytheroid-derived-2)-like 2
NSFT	novelty-suppressed feeding test
OFT	open-field test
PFC	prefrontal cortex
PGE2	prostaglandin E2
PPARγ	peroxisome proliferator-activated receptor
RA	retinoic acid
RAGE	Receptor for Advanced Glycation End-products
RIPK	receptor-interacting serine/threonine protein kinase
ROS	reactive oxygen species
SOD	superoxide dismutase
SPT	sucrose preference test
TLR4	Toll-like receptor 4
TrkB	tropomyosin receptor kinase B
UPLC-ESI-MS/MS	ultra-performance liquid chromatography–electrospray ionization–tandem mass spectrometry.
